# Phenotypic comparison between smoking and non-smoking chronic obstructive pulmonary disease

**DOI:** 10.1186/s12931-020-1310-9

**Published:** 2020-02-12

**Authors:** Sundeep S. Salvi, Bill B. Brashier, Jyoti Londhe, Kanchan Pyasi, Vandana Vincent, Shilpa S. Kajale, Sajid Tambe, Kuldeep Mandani, Arjun Nair, Sze Mun Mak, Sapna Madas, Sanjay Juvekar, Louise E. Donnelly, Peter J. Barnes

**Affiliations:** 1 Chest Research Foundation, Pune, India; 2 KEM Hospital Research Centre, Pune, India; 3 Department of Radiology, Royal Brompton Hospital, London, UK; 4 National Heart & Lung Institute, Imperial College, Dovehouse Street, London, SW3 6lY UK

**Keywords:** Chronic obstructive pulmonary disease, biomass smoke, non-smoking COPD, small airway disease, household air pollution

## Abstract

**Background:**

Although COPD among non-smokers (NS-COPD) is common, little is known about this phenotype. We compared NS-COPD subjects with smoking COPD (S-COPD) patients in a rural Indian population using a variety of clinical, physiological, radiological, sputum cellular and blood biomarkers.

**Methods:**

Two hundred ninety subjects (118 healthy, 79 S-COPD, 93 NS-COPD) performed pre- and post-bronchodilator spirometry and were followed for 2 years to study the annual rate of decline in lung function. Body plethysmography, impulse oscillometry, inspiratory-expiratory HRCT, induced sputum cellular profile and blood biomarkers were compared between 49 healthy, 45 S-COPD and 55 NS-COPD subjects using standardized methods. Spirometric response to oral corticosteroids was measured in 30 female NS-COPD patients.

**Results:**

Compared to all male S-COPD subjects, 47% of NS-COPD subjects were female, were younger by 3.2 years, had greater body mass index, a slower rate of decline in lung function (80 vs 130 mL/year), more small airways obstruction measured by impulse oscillometry (*p* < 0.001), significantly less emphysema (29% vs 11%) on CT scans, lower values in lung diffusion parameters, significantly less neutrophils in induced sputum (*p* < 0.05) and tended to have more sputum eosinophils. Hemoglobin and red cell volume were higher and serum insulin lower in S-COPD compared to NS-COPD. Spirometric indices, symptoms and quality of life were similar between S-COPD and NS-COPD. There was no improvement in spirometry in NS-COPD patients after 2 weeks of an oral corticosteroid.

**Conclusions:**

Compared to S-COPD, NS-COPD is seen in younger subjects with equal male-female predominance, is predominantly a small-airway disease phenotype with less emphysema, preserved lung diffusion and a slower rate of decline in lung function.

## Background

Chronic obstructive pulmonary disease (COPD) is the third leading cause of death worldwide, accounting for over 3 million deaths/year [1]. Over 90% of these deaths occur in low-income regions of the world, particularly in South Asia, South East Asia, Sub-Saharan Africa and South America [2]. Over 300 million people worldwide suffer from COPD, most of whom reside in low income countries [3].

Tobacco smoking has been an established risk factor for COPD for over five decades and virtually all our knowledge about the clinical, physiological, pathological and radiological features of COPD, as well as rates of decline in lung function and efficacies of various treatments is based on this population. In 1990, we reported that between 25 and 45% of COPD occurs among never smokers [4]. The 2017 Global Burden of Disease study has estimated that smoking accounts for only 35% of the global COPD burden, most of which occur in high income countries [1]. The remaining 65% of the non-smoking COPD burden occurs mostly in the low and middle income countries of the world. Exposure to biomass smoke during cooking in poorly ventilated homes, [5–7] high levels of ambient air pollution, occupational exposures to dust and gases, ambient ozone exposure, poverty, repeated respiratory tract infections during childhood, poorly-controlled chronic persistent asthma and previous tubercular lung disease are also non-smoking risk factors for COPD [8–10].

Despite the relatively high burden of COPD among never smokers, relatively little is known about this phenotype. In this study, we compared clinical, physiological, radiological, sputum cellular inflammatory profile and blood markers between smoking (S-COPD) and non-smoking COPD (NS-COPD) subjects and the spirometric response to a standard clinical trial of oral corticosteroids in NS-COPD females in order to better understand the similarities or differences between these two COPD phenotypes.

## Methods

### Study Participants

The study population included male and female subjects above the age of 40 years, who were recruited randomly from rural communities of Pune district in India. They were three groups: (i) Healthy: defined as otherwise fit and well subjects with no symptoms, no underlying diseases, no hospitalizations for any disease-related event in the past, normal clinical examination reported by a pulmonologist, normal chest X-ray and normal spirometry, (ii) S-COPD: tobacco smokers with at least 10 pack-years of cigarette or bidi smoking, who had no other respiratory diseases, such as asthma, based on history, clinical examination and chest X-ray findings, who had a post-bronchodilator FEV_1_/FVC ratio < 70% and FEV_1_ < 80% predicted, and (iii) NS-COPD: subjects exposed to biomass smoke for at least 2 h a day for 20 years and subjects exposed to occupational risk factors associated with COPD for at least 20 years, who had no other respiratory diseases based on history, clinical examination and chest X-ray findings, and who had a post-bronchodilator FEV_1_/FVC ratio of < 0.70 and FEV_1_ < 80% predicted. All NS-COPD patients were non-smokers and none of the subjects of COPD had a past or current history of asthma.

We screened 264 subjects in five lung health camps from across 22 villages near Pune in India and these provided 10 S-COPD and 14 NS-COPD subjects. We then randomly screened 468 households in the same geographic region, which provided an additional 69 S-COPD, 79 NS-COPD and 118 healthy subjects.

### Study Design

**Fig. 1:**
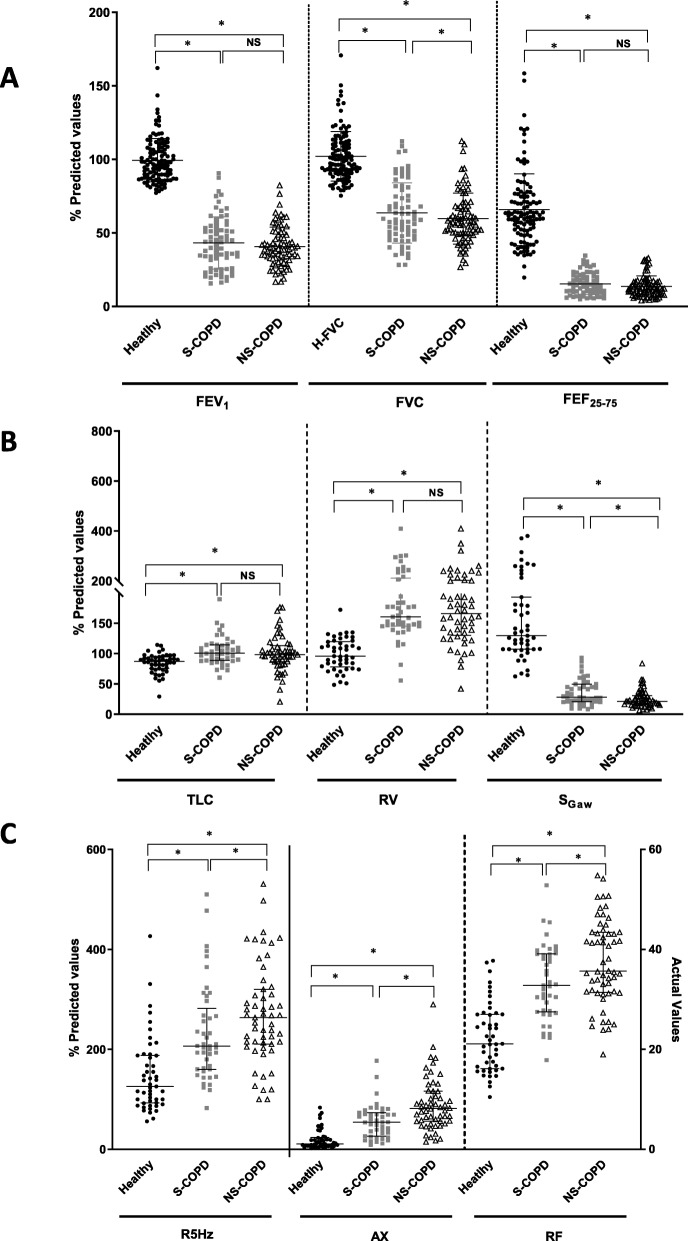
Differences in lung function between healthy (n = 114, ●), smoking (S)-COPD (*n* = 71, ■),) and non-smoking COPD (*n* = 82, △) using (**a**) spirometry, with forced expiratory volume in 1 s (FEV_1_), forced vital capacity (FVC) and forced expiratory flow between 75 and 25% of vital capacity (FEF_25–75_); **b** Lung volumes, showing total lung capacity (TLC), residual volume (RV), specific airway conductance (sG_aw_) and specific airway resistance (sR_aw_); **c** Impulse oscillometry, showing resistance at 5 Hz (R5), area under the reactance curve (AX), resonant frequency (RF). Data are presented as individual data points and the bars indicate median and interquartile ranges, where * indicates *p* < 0.05, NS = non-significant

The study was conducted in two parts: (1) a cross-sectional, case-control study that examined the phenotypic differences between S-COPD and NS-COPD subjects; (2) a longitudinal cohort study where healthy, S-COPD and NS-COPD subjects were followed over a period of 2 years. NS-COPD subjects were compared with S-COPD and healthy subjects. Further details of the methodology are provided in the (Fig. 1).

### Lung function

Pre- and post-bronchodilator spirometry, peak expiratory flow (PEF) and lung volumes were compared between healthy, S-COPD and NS-COPD subjects and measured, as described in the Online Supplement according to ATS/ERS standards [11]. Impulse oscillometry was performed to measure airway resistance, reactance and resonant frequency [12, 13].

### Imaging

Inspiratory-expiratory high-resolution computed tomography (HRCT) scans: were performed at full inspiration and then at full expiration, as described in the Online Supplement. HRCT scans were scored based on a modified version of previously validated scoring systems [14, 15].

### Induced sputum

Sputum was induced following nebulization with hypertonic saline and processed for measurement of total and differential cell counts using a standardized protocol [16].

### Other measurements

Height, weight, blood pressure and electrocardiogram were recorded in all subjects. Peripheral venous blood was collected and analyzed for serum insulin levels, hemoglobin, total cell counts, red blood cell counts, packed cell volumes and mean corpuscular volumes and high sensitivity C-reactive protein (hs-CRP). COPD symptoms were measured using the COPD Assessment Test (CAT) and respiratory quality of life by the St. George’s Respiratory Questionnaire (SGRQ) from the locally translated and validated versions of the questionnaires.

### Serial spirometry

The cohort of 290 study participants comprising 118 healthy, 79 S-COPD and 93 NS-COPD subjects who underwent the baseline pre and post-bronchodilator spirometry were followed up over a period of 2 years with annual measurements of pre and post-bronchodilator spirometry. Changes in symptoms and quality of life were also evaluated at baseline and every year for 2 years using CAT and SGRQ tests.

### Therapeutic response to oral prednisolone

In a proof-of-concept study, 30 randomly-selected NS-COPD female subjects (all exposed to biomass smoke) were invited to participate in this single-blind, placebo-controlled, interventional therapeutic trial with prednisolone 40 mg daily for 2 weeks (standard trial of steroids). We studied females with COPD associated with biomass smoke exposure as this was a well-defined NS-COPD group.

### Statistics

All demographic and other tests data were summarized using descriptive statistics as described in the Additional file 1.

## Results

### Study population

Table 1 shows the demographic characteristics of the study populations. The mean age of the NS-COPD subjects was significantly less than the S-COPD population. The S-COPD patients were all male, reflecting the fact that women in this population never smoke, whereas the NS-COPD patients were all non-smokers; 53% were male (exposed to occupational dust) and 47% female (all exposed to biomass smoke). The S-COPD subjects were exposed to 29.2 ± 22.9 (5.0–87.5) pack-years of smoking (29 out of the 117 subjects had between 5 and 10 pack years of smoking history, had no history of asthma and had spirometrically defined COPD), while the biomass smoke COPD subjects were exposed to 111.1 ± 66.3 (0.0–294) hour-years. As there were no significant differences between the NS-COPD patients with biomass smoke exposure compared to occupational exposure, these two groups were combined for subsequent analyses. Over 90% of the COPD population were receiving oral tablets (salbutamol and theophylline), 15% inhaled salbutamol, by 11% inhaled ipratropium and only 2% inhaled corticosteroid and long-acting β_2_-agonist combination. The 118 healthy controls who had normal spirometry included 27 healthy male smokers exposed to 25.5 ± 23.7 pack-years, 55 exposed to biomass smoke and 36 non-exposed.

**Table 1:** Subject demographics, lung function, symptom scores, imaging parameters, blood and sputum measurements

Variables	Healthy	S-COPD	NS-COPD (Biomass smoke)	NS-COPD (Occupational)^a^	S-COPD Vs. BS-COPD	S-COPD Vs. NS-COPD (Occupational)	BS-COPD Vs. NS-COPD (Occupational)
	Mean ± SD	Mean ± SD	Mean ± SD	Mean ± SD			
N	118	79	59	34			
Sex (M: F)	53%: 47%	100%: 0%	0%: 100%	100%: 0%			
Age (Years)	64.4 ± 7.8	67.2 ± 7.4	62.4 ± 7.3	66.7 ± 8.1	< 0.0001	p = ns	0.010
BMI (kg/m^2^)	21.9 ± 3.8	19.3 ± 3.4	21.3 ± 4.4	20.0 ± 3.3	0.002	p = ns	p = ns
Spirometry							
FEV1 Pre (% Predicted)	99.4 ± 14.7	43.1 ± 17.8	39.9 ± 12.4	41.9 ± 14.4	p = ns	p = ns	p = ns
FVC Pre (% Predicted)	102.1 ± 16.8	63.6 ± 20.5	57.9 ± 16.04	62.6 ± 19.2	0.005	0.732	0.032
Δ Reversibility FEV1 Absolute (mL)	40.0 ± 93	160.0 ± 154.0	160 ± 130	170 ± 190	p = ns	p = ns	p = ns
Δ Reversibility FEV1%	2.2 ± 4.9	19.0 ± 19.9	25.6 ± 24.1	19.3 ± 20.6	p = ns	p = ns	p = ns
Δ Reversibility FVC Absolute (mL)	0.0 ± 190.0	237.0 ± 312.0	240 ± 230	290 ± 300	p = ns	p = ns	p = ns
Δ Reversibility FVC %	0.3 ± 7.4	15.5 ± 22.6	23.2 ± 31.5	18.2 ± 22.2	p = ns	p = ns	p = ns
FEV1/FVC Post bronchodilator (%)	80.5 ± 6.1	53.1 ± 10.9	56.4 ± 8.2	52.8 ± 9.9	0.045	p = ns	0.051
PEF (% Predicted)	89.2 ± 16.0	34.5 ± 15.8	29.8 ± 11.2	34.9 ± 24.3	p = ns	p = ns	p = ns
FEF25–75 Pre % Predicted	65.7 ± 24.4	15.3 ± 7.4	12.8 ± 6.9	14.8 ± 7.4	p = ns	p = ns	p = ns
Plethysmography							
TLC Pre (% Predicted)	84.2 ± 15.9	104.1 ± 23.1	99.8 ± 32.6	103.2 ± 27.3	p = ns	p = ns	p = ns
RV Pre (% Predicted)	98.8 ± 26.4	180.8 ± 65.09	176.2 ± 72.1	174.2 ± 59.6	p = ns	p = ns	p = ns
IC Pre (% Predicted)	75.2 ± 27.3	49.6 ± 18.7	49.8 ± 23.7	43.7 ± 19.3	p = ns	p = ns	p = ns
RV/TLC Pre (% Predicted)	116.8 ± 21.4	171.5 ± 29.7	170.9 ± 39.4	175.7 ± 31.2	p = ns	p = ns	p = ns
SGaw Pre (% Predicted)	159.6 ± 78.0	36.2 ± 21.0	24.5 ± 13.6	26.9 ± 16.3	0.010	p = ns	p = ns
IOS							
R5 (kPa s L^−1^) Pre (% Predicted)	148 ± 74.8	231.8 ± 96.4	287.3 ± 100.1	244.2 ± 93.5	0.005	p = ns	p = ns
R20 (kPa s L^− 1^) Pre (% Predicted)	118.0 ± 50.6	130.1 ± 44.6	158.3 ± 51.2	134.6 ± 47.1	0.012	p = ns	p = ns
R5-R20 (kPa s L^− 1^) Pre	0.16 ± 0.1	0.36 ± 0.2	0.60 ± 0.3	0.39 ± 0.2	< 0.0001	p = ns	0.003
X5 Pre (kPa s L^− 1^)	−0.24 ± 0.2	− 0.56 ± 0.3	−0.9 ± 0.4	−0.7 ± 0.3	< 0.0001	p = ns	p = ns
Ax Pre (Hz kPa s L^− 1^)	1.96 ± 2.0	5.59 ± 3.5	10.3 ± 5.7	7.1 ± 3.7	< 0.0001	p = ns	0.034
RF Pre (Hz)	22.4 ± 6.9	33.4 ± 7.5	37.2 ± 7.4	38.1 ± 10.0	0.022	0.041	p = ns
Gas Diffusion							
DLCo % Predicted	76.7 ± 17.3	54.2 ± 21.9	54.7 ± 17.3	70.2 ± 27.0	p = ns	p = ns	p = ns
DLCO / VA	102.5 ± 17.6	79.9 ± 30.3	83.5 ± 15.9	96.2 ± 32.4	p = ns	p = ns	p = ns
HRCT							
Normal (%)	4.3%	0%	2.4%	0%			
Airways Disease Predominant (%)	59.6%	60.5%	95.2%	71.4%			
ILD Predominant (%)	34.0%	10.5%	0%	3.6%			
Emphysema Predominant (%)	2.1%	28.9%	1 (2.4%)	25.0%s			
Emphysema Score	19.9 ± 38.9	265 ± 342	39.6 ± 69.9	193.4 ± 313.9	0.001	p = ns	0.006
↓attenuation on the expiratory CT Score	164.3 ± 169.7	278.9 ± 237.8	479.5 ± 232.7	422 ± 329.1	0.001	0.024	p = ns
CAT Score		23.6 ± 7.8	22.0 ± 6.5	25.2 ± 8.8	p = ns	p = ns	p = ns
SGRQ Score		63.5 ± 18.5	60.2 ± 18.4	59.7 ± 16.3	p = ns	p = ns	p = ns
CRP (mg/L)	2.3 ± 2.9	3.6 ± 4.6	5.2 ± 6.7	4.3 ± 3.9	p = ns	p = ns	p = ns
Serum Insulin (μIU/mL)	5.5 ± 3.2	3.7 ± 2.7	8.8 ± 4.7	5.3 ± 5.6	0.008	p = ns	p = ns
Hemoglobin (mg/dL)	12.8 ± 1.2	13.6 ± 2.3	11.9 ± 1.9	13.0 ± 2.0	< 0.0001	p = ns	p = ns
Red cell count (10^6^ cells/μL)	4.3 ± 0.5	4.6 ± 0.6	4.3 ± 0.4	4.6 ± 0.5	0.007	p = ns	p = ns
Packed cell volume (%)	39.6 ± 5.2	41.8 ± 6.4	36.7 ± 5.3	40.1 ± 5.8	< 0.0001	p = ns	0.051
Mean corpuscular volume (fL)	92.4 ± 11.2	91.4 ± 10.8	84.7 ± 10.8	88.2 ± 9.8	0.004	p = ns	p = ns
Blood eosinophils (%)	4.6 ± 3.9	4.7 ± 4.7	5.3 ± 4.7	4.6 ± 4.43	p = ns	p = ns	p = ns
Blood eosinophils (cells/μL)	330.4 ± 319	343.5 ± 408	410.03 ± 403.73	337.5 ± 330.09	p = ns	p = ns	p = ns
Sputum							
Total cell count per ml	1.38 (0.9 2.9)	6.19 (3.2, 9.3)	4.51 (2.5, 6.9)	3.6 (2.4, 7.4)	p = ns	p = ns	p = ns
Macrophages per ml	0.33 (0.2, 0.6)	0.87 (0.4, 1.2)	0.72 (0.4, 1.3)	0.60 (0.3, 0.9)	p = ns	p = ns	p = ns
Neutrophils per ml	0.87 (0.5, 2.8)	5.22 (2.6, 8.3)	2.63 (1.7, 5.9)	2.60 (1.2, 6.7)	p = ns	p = ns	p = ns
Eosinophils per ml	0.006 (0.0, 0.02)	0.139 (0.04, 0.3)	0.25 (0.04, 0.4)	0.21 (0.1, 0.6)	p = ns	p = ns	p = ns
Lymphocytes per ml	0.014 (0.0, 0.03)	0.032 (0.02, 0.08)	0.03 (0.01, 0.07)	0.03 (0.01, 0.05)	p = ns	p = ns	p = ns

^a^Out of the 34 occupational COPD, 25 were farmers, 4 were farmers + hard rock mining/sandblasting exposure and 5 had occupational dust exposures for at least 20 years

*FEV*_*1*_ Forced expiratory volume in 1 s, *FVC* Forced vital capacity, *FEF*_*25–75*_ Forced expiratory flow between 25 and 75% of FVC, *PEF* Peak expiratory flow, *BD* Bronchodilator, *p-y* Pack-years, *h-y* hour-years, *sRaw* specific airway resistance, *sGaw* specific airway conductance, *TLC* Total lung capacity, *RV* Residual volumeM, *IOS* Impulse oscillometry, *R5* Resistance at 5 Hz, *X5* Reactance at 5 Hz, *AX* Area under the reactance curve, *Fres* Resonant frequency, *D*_*LCO*_ Diffusing lung co-efficient for carbon monoxide, *K*_*CO*_ Transfer coefficient, *SGRQ* St. George’s Respiratory Questionnaire, *CAT* COPD assessment test, *hsCRP* high sensitivity C-reactive protein

Data are presented as means ± SD

### Spirometry

FEV_1_, FVC, FEV_1_/FVC, PEF and FEF_25–75%_ values were all significantly lower among both S-COPD and NS-COPD subjects compared to age-adjusted healthy subjects (Fig. 1a, Table 1). NS-COPD subjects had lower FVC values than S-COPD (statistically significant, but clinically very small), but no differences were observed for other spirometric parameters between the two COPD groups. However, bronchodilator reversibility, as defined by > 12% and > 200 mL change in FEV_1_ after inhaled salbutamol (200 μg), was seen in 39.2% of S-COPD and 33.7% of the NS-COPD subjects.

### Plethysmography

NS-COPD subjects had significantly higher sRaw values than S-COPD subjects (*p* = 0.005, Table 1); sGaw values were significantly lower among both S-COPD (*p* < 0.0001) and NS-COPD subjects (*p* < 0.0001) compared to healthy subjects. NS-COPD subjects had significantly lower sGaw than S-COPD (*p* = 0.006, Fig. 1b, Table 1). Residual volumes, total lung capacities and RV/TLC ratios were significantly increased among both S-COPD and NS-COPD subjects compared to healthy subjects (*p* < 0.0001), with no differences between the two COPD groups.

### Impulse oscillometry

Resistance at 5 Hz (R5), area under the reactance curve (Ax) and resonant frequency (Rf) values were significantly greater in NS-COPD than S-COPD subjects (Fig. 1c, Table 1), but there were no differences in R5-R20, R5-R20/R5 and reactance at 5 Hz (X5). When we split the NS-COPD into biomass smoke and occupational exposures, biomass smoke exposed subjects showed a significantly greater increase in R5-R20 values compared to occupationally exposed subjects (p < 0.0001). Occupationally exposed COPD subjects showed similar values to S-COPD subjects.

### Gas diffusion

Single-breath D_LCO_ values divided by alveolar volume (D_LCO_/V_A_) were significantly lower among both S-COPD (*p* = 0.001) and NS-COPD (*p* = 0.04) subjects compared to healthy subjects with no significant differences between the two groups, although numerically NS-COPD subjects had higher D_LCO_/V_A_ values (Table 1).

### HRCT scans

**Fig. 2:**
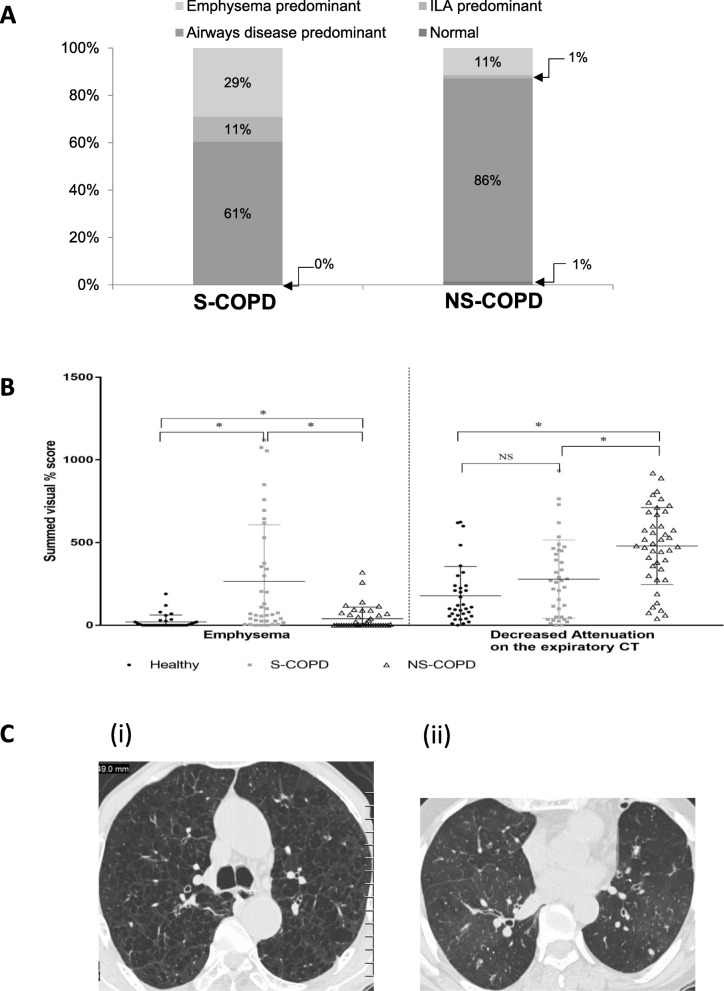
Differences between smoker COPD (S-COPD) (*n* = 38) and non-smoker COPD (NS-COPD) (*n* = 70) on inspiratory-expiratory high resolution computerized tomography (HRCT) imaging (a) Showing HRCT classification of smoker and non-smoker COPD, including airway disease (black bars), emphysema (open bars) and interstitial lung abnormality (ILA) predominance (grey bars); (b) dot plot for emphysema and decreased attenuation of expiratory CT in S-COPD (■) and NS=COPD (△) compared to healthy subjects (●), where bars indicate median and interquartile rages and * *p* < 0.05, (c) Representative HRCT images of Smoker-COPD showing extensive centrilobular emphysema in the upper lobes and non-smoker COPD showing generalized decreased attenuation and some bronchial wall thickening

Inspiration-expiration HRCT scans showed that in S-COPD subjects, 29% had emphysema, 10% had respiratory bronchiolitis interstitial lung disease (RB-ILD)-type pattern and 61% had airways disease predominant pattern. None of the S-COPD subjects in our study had a normal HRCT. Among the NS-COPD subjects, only 11% had emphysema, 86% had airways disease predominant pattern and 1% had normal HRCT (Fig. 2a). The emphysema score was significantly greater among S-COPD subjects than NS-COPD subjects (265 ± 342% vs 39.6 ± 69.9%; *p* = 0.004). In contrast, the decreased attenuation on expiratory CT values (mean ± SD) were significantly greater in NS-COPD subjects than S-COPD subjects (479.4 ± 232.6& vs 278.9 ± 237.8%; *p* = 0.04) (Fig. 2b). Representative HRCT images of S-COPD and NS-COPD are shown in Fig. 2c.

### Symptoms

NS-COPD subjects had similar CAT and SQRQ scores (including activity and impact scores) to S-COPD subjects (Table 1).

### Sputum analysis

**Fig. 3:**
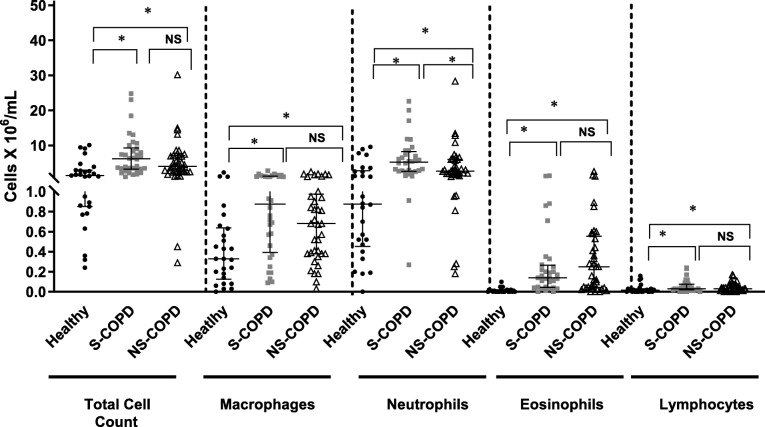
Inflammatory cell counts in induced sputum from healthy controls (*n* = 27, ●), smoking (S)-COPD (n = 34, ■) and non-smoking (NS)-COPD (*n* = 37, △). Data are presented as individual data points and bars indicate median and interquartile ranges, * = *p* < 0.05, **p < 0.01

Total sputum cell counts were significantly greater among both S-COPD and NS-COPD compared to healthy subjects (Fig. 3), with no differences between the two groups. Total neutrophil counts were significantly greater in the S-COPD (*p* < 0.0001) and NS-COPD subjects (*p* = 0.004) compared to healthy subjects. However, S-COPD subjects had greater numbers of neutrophils than NS-COPD subjects (*p* = 0.04). Eosinophil counts were significantly greater among both S-COPD (*p* < 0.0001) and NS-COPD *P* < 0.0001) compared to healthy subjects. Although NS-COPD subjects had 1.4-times more sputum eosinophils than S-COPD subjects, the difference was not statistically significant. Macrophage and lymphocyte counts were significantly greater amongst both COPD groups compared to healthy subjects, and no differences were found between the two COPD groups.

### Systemic measurements

S-COPD subjects had significantly lower body mass index (BMI) than NS-COPD (p = 0.009) and healthy subjects (*p* < 0.0001, Table 1). NS-COPD subjects had significantly greater hsCRP levels in serum than healthy subjects and no difference from S-COPD subjects. S-COPD subjects had significantly lower serum insulin levels than healthy subjects, while NS-COPD subjects had significantly higher insulin levels than S-COPD subjects. S-COPD subjects had higher blood haemoglobin, red blood cell counts, packed cell volume and mean corpuscular volume compared to NS-COPD subjects. There were no differences circulating eosinophil counts between S-COPD and NS-COPD subjects.

### Clinical trial of oral steroids

**Fig. 4:**
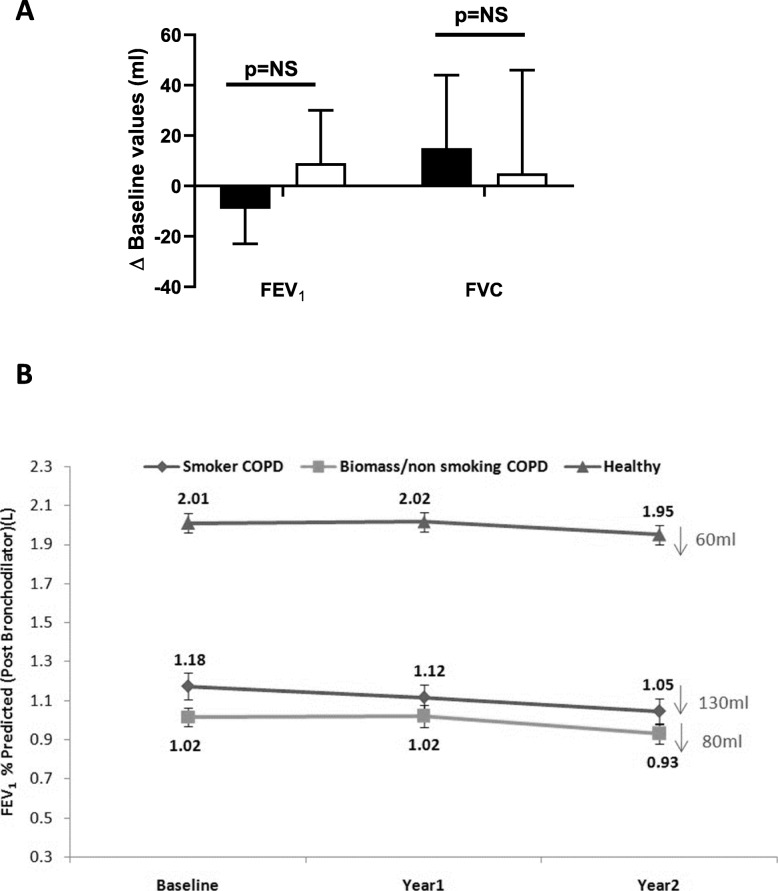
Panel (**a**) Change in FEV_1_ and FVC after 4 weeks of oral prednisolone (30 mg/day) in female non-smoking COPD patients (open bars) or placebo (black bars); data are mean values ± SEM; NS = non-significant; Panel (**b**) Decline in post-bronchodilator FEV_1_ between healthy (*n* = 88), smoking COPD (*n* = 44) and non-smoking COPD subjects (*n* = 62) over 2 years

Thirty female NS-COPD subjects (mean age: 63.7 ± 7.8 years, baseline % predicted (ECCS values with 0.9 correction) FEV_1_: 41.0 ± 13.9, baseline % predicted FVC: 65.0 ± 15.9, FEV_1_ reversibility: 180 mL ± 100 mL, 28.1% ± 15.7%) completed the study. Oral prednisolone (30 mg once daily) for 4 weeks did not result in any significant improvements in pre-bronchodilator FEV_1_ (9 ± 21 mL; p = ns) or FVC (5 ± 41 mL; p = ns) values (Fig. 4a).

### Longitudinal follow-up

Over a period of 2 years, post-bronchodilator FEV_1_ values reduced by 130 ± 37 mL in S-COPD subjects and 80 ± 30 mL in NS-COPD subjects (Fig. 4b). Total SGRQ scores did not change in either S-COPD or NS-COPD subjects over this period (S-COPD: 63.0 ± 21.0 after 1 year and 59.9 ± 19.9 after 2 years; NS-COPD: 60.1 ± 18.5 after 1 year and 61.5 ± 20.5 after 2 years; p = non-significant by repeated measures analysis). No significant differences were seen in either COPD group in symptoms, activity and impact scores over the 2-year follow-up. Similarly, CAT scores did not change significantly over 2-years in either COPD group compared to baseline.

## Discussion

Tobacco smoking accounts for only 35% of the population attributable risk for COPD, with the remaining risk attributed to ambient air pollution, household air pollution, occupational exposures and exposure to ozone among several other risk factors [1, 2, 6]. Despite the high burden of NS-COPD, relatively little is known about this phenotype [4]. We believe that this is the first study that has examined the phenotypic differences between S-COPD and NS-COPD subjects within the same population in a comprehensive manner using a wide range of clinical, physiological, radiological, systemic and sputum cellular inflammatory parameters. We report some similarities, but also differences between S-COPD and NS-COPD.

Strong sex differences were observed between S-COPD and NS-COPD subjects (100% vs 53% males respectively), because tobacco smoking among rural Indian women is very rare (<1%). Women spend most of their time cooking food using biomass fuels and are therefore at a greater risk of developing biomass-smoke exposed NS-COPD, whereas male NS-COPD subjects were exposure to occupational dust. Because there were sex differences between S-COPD and NS-COPD, we used % predicted values for physiological comparisons which adjusted for age, sex, height and weight. For a similar degree of airflow obstruction measured by FEV_1_% predicted (S-COPD: 43.1% vs NS-COPD: 40.7%), 36 pack years of tobacco smoking were equivalent to 46 years of exposure to biomass smoke (average of 2 h daily), which suggests that 10 pack-years of tobacco smoking is equivalent to 13 years of exposure to household biomass smoke.

The only spirometric difference between S-COPD and NS-COPD subjects was a lower FVC among NS-COPD subjects. Although the difference was statistically significant, clinically it was only very marginal. Lower FVC values have also been reported among Mexican COPD women exposed to biomass smoke compared to those who had COPD due to tobacco smoking [17]. while an earlier necropsy study in women with COPD due to exposure to wood smoke showed more small airway fibrosis than in S-COPD [18]. Impulse oscillometry using the forced oscillation technique is a more sensitive and specific tool to study small airway function [12, 19]. Significantly greater AX and Rf values were observed among NS-COPD subjects, suggesting a greater degree of ventilation heterogeneity, which may indicate greater small airways obstruction than in S-COPD. Peripheral airway compliance as area under the reactance curve (AX) was increased to a greater extent in NS-COPD than in S-COPD. An increased Ax represents a lower apparent respiratory compliance, which apart from small airway narrowing [12], could also be due to changes in tissue properties, which would manifest as interstitial change or increase in mean lung density. D_LCO_ values were numerically lower amongst S-COPD than NS-COPD subjects, but these differences were not statistically significant. Others have reported significantly greater reduction in D_LCO_ values among smoker COPD subjects [20]. The inspiratory and expiratory HRCT scans further support this difference as more S-COPD than NS-COPD subjects had emphysema. In contrast, NS-COPD subjects had more airways disease-predominant pattern and a 1.7-fold greater decrease in expiratory lung attenuation scores, indicating greater small airways obstruction and gas trapping. Taken together, these observations suggest that NS-COPD is predominantly a small airways disease phenotype with less emphysema than S-COPD in agreement with previous smaller studies [17, 21–23].

Despite the significant differences between S-COPD and NS-COPD in lung function tests and radiology, the quality of life and symptoms as measured by SGRQ and CAT scores between the two groups were similar. Interestingly, the mean SGRQ and CAT scores in our study population are around 50% greater than the average scores reported in studies from Western nations for a similar degree of FEV_1_ impairment (~ 40% predicted), suggesting that both S-COPD and NS-COPD subjects in India have a worse quality of life than in high income countries.

In our study, S-COPD subjects had a significantly lower BMI than NS-COPD subjects, an observation also been reported in Mexico [24] and Spain [21]. Compared to healthy subjects, serum insulin levels were 37% lower in S-COPD and 15% higher in NS-COPD. Tobacco smoking impairs insulin secretion in a dose-dependent manner [25], possibly mediated by nicotine-induced apoptosis of pancreatic beta-cells [26]. We speculate that the lower body weight among S-COPD subjects could be due to defective insulin secretion, which does not seem to occur in NS-COPD subjects, but this observation needs more study.

Induced sputum total inflammatory cell numbers between S-COPD and NS-COPD subjects were similar, but neutrophil numbers were higher in S-COPD subjects, while eosinophil numbers tended to be higher among NS-COPD subjects. In the peripheral blood, mean absolute eosinophil counts were 16% higher in NS-COPD subjects than S-COPD subjects, although not statistically significant. Higher eosinophil counts in the sputum and circulating blood of NS-COPD subjects might suggest that they might have a better therapeutic response to corticosteroids [27, 28]. However, we found that female NS-COPD subjects with a baseline FEV_1_ of 40% predicted and a reversibility of 180 mL and 28%, when treated for 4 weeks with high dose oral corticosteroid did not show any significant improvements in spirometry, demonstrating a similar corticosteroid-resistance to that seen in S-COPD patients [29]. This observation argues against an asthmatic component to this phenotype of COPD, although more studies with inhaled corticosteroids need to be conducted before any conclusive statement can be made.

The rate of decline in post-bronchodilator FEV_1_ over 2 years was slower in NS-COPD than S-COPD (80 mL vs 130 mL). Similar observations have been reported among Mexican COPD subjects [24]. The slower rate of decline in FEV_1_ observed among NS-COPD subjects could be explained by several factors; (a) a lower starting baseline value (1.02 L vs 1.18 L) and therefore a smaller absolute decline, (b) smaller lungs because of early life and chronic exposures to biomass smoke, and (c) less emphysema, whose presence has been shown to be a strong determinant of rapid decline in lung function [30]. We agree that 2 years is a relatively small duration to look for long-term declines in rate of lung function and this is a limitation of our study.

## Conclusions

Compared to S-COPD subjects, NS-COPD subjects are more likely to be younger, female, have a greater BMI, more small airways obstruction and less emphysema, less impairment in gas diffusion, less neutrophils and more eosinophils in sputum and a slower rate of decline in lung function. Spirometry indices and quality of life are similar between S-COPD and NS-COPD and there does not appear to be any improvement with oral corticosteroids, as with S-COPD patients. Future studies should investigate which therapeutic interventions are most appropriate for NS-COPD and whether they manifest with the same co-morbid conditions as S-COPD subjects.

## Supplementary information

**Additional file 1 Methods S1.**

## Abbreviations

AX: Area under the reactance curve
BMI: Body mass index
CAT: COPD assessment test
CRP: C-reactive protein
D_LCO_: Diffusing lung co-efficient for carbon monoxide
FEF_25–75_: Forced expiratory flow between 25 and 75% predicted of FVC
FEV_1_: Forced expiratory volume in 1 s
Fres: Resonant frequency
FVC: Forced vital capacity
HRCT: High resolution computed axial tomography
ILA: Interstitial lung abnormality on HRCT
IOS: Impulse oscillometry
NS-COPD: Non-smoking COPD
PEF: Peak expiratory flow
R5: Resistance at 5 Hz;
RV: Residual volume
S-COPD: Smoking COPD
sGaw: specific airway conductance
SGRQ: St. George’s Respiratory Questionnaire
sRaw: specific airway resistance
TLC: total lung capacity
V_A_: Alveolar volume
X5: Reactance at 5 Hz
