# Supplementary material for: Phenotypic comparison between smoking and non-smoking chronic obstructive pulmonary disease
Source: Respir Res. 2020 Feb 12;21:50. doi: 10.1186/s12931-020-1310-9 (PMC7017521; doi:10.1186/s12931-020-1310-9)
Supplement: Supplementary file 1 — Additional file 1 Methods S1. [file 12931_2020_1310_MOESM1_ESM.docx]

**Additional file 1**

**S1. Methods**

**Spirometry**

Pre- and post-bronchodilator spirometry was performed using an Ultrasonic Spirometer (Medical Technologies Inc., Andover, MA) according to the American Thoracic Society/European Respiratory Society 2005 recommendations. FEV_1_, FVC, forced expiratory flow between 25% to 75% of the FVC (FEF_25-75%_) and peak expiratory flow (PEF) values were compared between healthy, S-COPD and NS-COPD subjects. From the pool of 118 healthy, 79 S-COPD and 93 NS-COPD subjects that underwent spirometry, we randomly selected 50 each from the healthy and S-COPD groups and 70 from NS-COPD group to further undergo the following tests:

**Body Plethysmography**

Lung volumes were measured by body plethysmography was performed using the Jaeger Bodyscreen bodybox (Jaeger, Wurzburg, Germany) according to ATS guidelines. Specific airway resistance (sRaw), specific airway conductance (sGaw), residual volume (RV) and total lung capacity (TLC) values were measured and compared between the study groups.

**Impulse oscillometry**

Impulse oscillometry was performed using the forced oscillation technique (Jaeger, Wurzburg, Germany). Resistance values at 5 Hz (R5) and 20 Hz (R20), reactance values at 5 Hz (X5) and 20 Hz (X20), area of reactance (Ax) and resonant frequency (Rf) values were measured and compared between the study groups.[12, 13]

**Inspiratory-expiratory HRCT scans**

All HRCT scans were performed using a helical 16-slice Philips Brilliance CT scanner (Philips Healthcare, Best, Netherlands). Interspaced acquisition from the thoracic apex to the diaphragm was performed at full inspiration followed by end-expiration, both at 120 kVp and 200 mAs with scan acquisition time of 0.5s. Axial datasets were reconstructed at 0.8mm thickness and 10mm intervals using a lung algorithm. The evaluation of HRCT images was performed by the Department of Thoracic Imaging at the Royal Brompton Hospital, London. Images were reviewed on appropriate lung windows (window level -500 HU, window width 1500 HU). The CTs were evaluated independently by two experienced radiologists with no access to clinical information. Discrepancies, if any, were resolved through consensus.

Several morphological features were visually evaluated in all 6 lobes (the lingula was considered a separate lobe). The lobar extent/severity of the following HRCT abnormalities were scored semi-quantitatively:

1. the extent of bronchiectasis (0=no bronchiectasis; 1=bronchiectasis in one or part of one bronchopulmonary segment; 2=bronchiectasis in more than one bronchopulmonary segment and 3=generalised bronchiectasis);
2. the severity of bronchiectasis (0=no airway dilatation; 1=100-200% of the transverse diameter of the homologous pulmonary artery and 2=more than 200-of the transverse diameter of the homologous pulmonary artery);
3. the severity of bronchial wall thickening (0=no discernible wall thickening; 1 =trivial wall thickening; 2= generally less than 25% of the transverse diameter of the homologous pulmonary artery; 3= greater than 25% of the transverse diameter of the accompanying pulmonary artery);
4. the extent of tree-in-bud nodularity (0=no plugging; 1=mild plugging, approximately <20% of the lobe; 2= moderate plugging, approximately 20-55% of the lobe; 3= severe plugging, approximately >55% of the lobe); and
5. the extent of consolidation (0=none; 1=mild, approximately <20% of the lobe; 2= moderate, approximately 20-55% of the lobe; 3= severe, approximately >55% of the lobe);

For these semi-quantitative findings, a greater than one grade difference for semi-quantitatively scored patterns was resolved by consensus between the observers, except for consolidation, where any degree of difference was scored (as this pattern itself was rare).

Following semi-quantitative evaluation, the following parenchymal patterns were classified in each lobe:

1. the overall extent of interstitial lung abnormality in each lobe, to the nearest 5% of the lobar volume, and the proportional extents of the following HRCT patterns within the interstitial abnormality (totaling 100%): a) respiratory-bronchiolitis-type nodularity ; b) a reticular pattern, c) ground-glass opacification, and d) honeycombing;
2. Emphysema extent, to the nearest 5% of the lobar volume; and
3. Decreased attenuation reflective of air-trapping, comparing inspiratory and expiratory scans, (and excluding areas already defined as emphysema), to the nearest 5% of the lobar volume.

The threshold for consensus for discrepant parenchymal patterns was a difference of greater than 10% for interstitial abnormality and emphysema, a difference of greater than 25% for decreased attenuation, and difference of any magnitude for the constituent interstitial patterns of respiratory-bronchiolitis-type nodularity, reticular pattern, ground-glass opacification, and honeycombing (as these were rare).

The semi-quantitative scores and percentages were summed across all 6 lobes and both readers. For example:

- a patient scored as grade 1 bronchiectasis extent in each lobe by each radiologist would have a total bronchiectasis extent score of (1 x 6 + 1 x 6)=12;
- a patient scored as 25% decreased attenuation extent in each lobe by one radiologist and 30% decreased attenuation extent in each lobe by the other radiologist would have a total decreased attenuation score of (25 x 6 + 30 x 6)=330%,

The CT of each individual was further categorized as one of the following patterns: 1) normal, 2) airways disease-predominant, 3) interstitial lung abnormality (ILA)-predominant or 4) emphysema-predominant

**Therapeutic response to oral prednisolone**

In a proof-of-concept study, 30 randomly-selected NS-COPD female subjects were invited to participate in this single-blind, placebo-controlled, interventional therapeutic trial with a high dose of oral corticosteroids (trial of steroids). Study participants received matched placebo tablets once daily in the morning after breakfast for 4 weeks. Pre-bronchodilator spirometry were measured at baseline and after the placebo treatment period. This was followed by an active treatment period of 4 weeks with oral prednisolone (30 mg), which was given once daily in the morning after breakfast. Pre-bronchodilator spirometry values were measured at baseline and after 4 weeks of treatment.

**Statistics**

The demographic, lung function and symptoms data have been summarized in Table 1. We presented categorical variables as frequencies and percentages, and analysed statistical differences using χ^2^ test or Fisher exact test when required. We presented continuous variables as N, Mean ± SD or Median (IQR) depending on the distribution of the data. Normality test (one sample Kolmogorov Smirnov test) was performed for all the parameters before applying any statistical hypothesis tests. Parametric tests were used for normally distributed data and non-parametric tests were used for non-normal data. Since there was significant difference in demographic characteristics between study groups, ANCOVA with post-hoc analysis was used to compare mean differences between different study groups, namely Healthy, S-COPD, BS-COPD and NS-COPD. The mean differences were compared after adjusting for all confounding factors such as age and BMI for all the data in Table 1. For non-normally distributed, the non-parametric Kruskal-Wallis tests was applied for comparing differences between different study groups Post hoc test for non-normally distributed data was performed using Mann-Whitney test. Data has been represented by median values in the figures. Repeated measures of analysis was used to analyze the decline in lung function over the period of 2 years. Statistical significance was reported at the p<0.05 level (two-sided significance). All the statistical computation was performed using SPSS 22.0 version.
